# Associations among Adolescents’ Relationships with Parents, Peers, and Teachers, Self-Efficacy, and Willingness to Intervene in Bullying: A Social Cognitive Approach

**DOI:** 10.3390/ijerph17020420

**Published:** 2020-01-08

**Authors:** Sebastian Wachs, Anke Görzig, Michelle F. Wright, Wilfried Schubarth, Ludwig Bilz

**Affiliations:** 1 Department of Educational Studies, University of Potsdam, 14476 Potsdam, Germany; wilschub@uni-potsdam.de; 2 Department of Psychology, University of West London, 310 Paragon House, Brentford TW8 9GA, UK; anke.gorzig@uwl.ac.uk; 3 Department of Psychology, Pennsylvania State University, University Park, State College, PA 16802, USA; mfw5215@psu.edu; 4 Faculty of Social Studies, Masaryk University, 60200 Brno, Czech Republic; 5 Institute of Health Sciences, Brandenburg University of Technology Cottbus-Senftenberg, 01968 Senftenberg, Germany; ludwig.bilz@b-tu.de

**Keywords:** bullying, intervention, willingness to intervene, bullying victimization, school, parent–child relationship, teacher–student relationship, self-efficacy

## Abstract

We applied the Social Cognitive Theory to investigate whether parent–child relationships, bullying victimization, and teacher–student relationships are directly as well as indirectly via self-efficacy in social conflicts associated with adolescents’ willingness to intervene in a bullying incident. There were 2071 (51.3% male) adolescents between the ages of 12 and 17 from 24 schools in Germany who participated in this study. A mediation test using structural equation modeling revealed that parent–child relationships, bullying victimization, and teacher–student relationships were directly related to adolescents’ self-efficacy in social conflicts. Further, teacher–student relationships and bullying victimization were directly associated with adolescents’ willingness to intervene in bullying. Finally, relationships with parents, peers and teachers were indirectly related to higher levels of students’ willingness to intervene in bullying situations due to self-efficacy in social conflicts. Thus, our analysis confirms the general assumptions of Social Cognitive Theory and the usefulness of applying its approach to social conflicts such as bullying situations.

## 1. Introduction

Bullying describes any repeatedly aggressive behavior against individuals or groups of people that feel or actually are powerless to defend themselves [1]. Bullying can have various forms such as physical bullying (i.e., hitting, kicking, and pushing), verbal bullying (i.e., hurtful nicknames and verbal insults), and relational bullying (i.e., manipulation, damaging the social standing within a peer group, and exclusion from peer activities) [2]. Verbal and relational bullying are of special concern because, contrary to popular belief, past research has shown that verbal bullying is the most prevalent form and relational bullying the most damaging form of bullying for adolescents’ development [1,3]. Thus, we will focus in the present study on these both forms of bullying.

Some research has shown that bullying rarely happens without witnesses [4]. Students most often experience bullying by witnessing it as bystanders [5]. A bystander is an individual who witnesses bullying but is not actively involved [6]. Bystanders initially show a tendency to remain passive due to various factors, such as being afraid of being bullied themselves, fear of judgement by peers, a diffusion of responsibility among the peer group at large, or a feeling of not possessing adequate skills for intervening [7]. However, the active involvement of students in support of the victim is linked with the termination of bullying events, impacts the prevalence of bullying incidents as a whole, contributes largely to the success of anti-bullying programs, and influences the positive adjustment and social status of the victim [6,8,9,10]. Moreover, witnessing bullying incidents negatively impacts the stress levels, emotions, school satisfaction, and mental health of bystanders; hence, assuming an active role may benefit not only the victim but bystanders also [11,12,13]. When witnessing bullying incidents, bystanders can feel co-victimized as well as experience cognitive dissonance between their actual and their desired behavioral response [11]. Thus, addressing bystanders’ active involvement as defenders of victims has become a crucial part of successful prevention and intervention programs [14].

In the current research, we apply Social Cognitive Theory (SCT) by Bandura [15] to the role of social relationships with primary socialization agents as well as an individual’s self-efficacy beliefs for the willingness to intervene in bullying. Here, we are situating SCT within a wider socio-ecological perspective [16] and the socio-ecological framework of bullying in particular [17], which emphasize the influence of various contexts in the social environment on human behavior. We focus on the primary socialization agents suggested by SCT across various environmental contexts (i.e., family, peers and school). Whilst the socio-ecological approach emphasizes the general influence of contextual factors on an individual’s development, it does not specify the directions or mechanisms for developmental or behavioral outcomes. In line with SCT, we focus here on self-efficacy as the primary mechanism for human agency.

SCT posits that learning occurs via processes of modeling and imitation [15]. The central role models put forward by SCT are the primary socialization agents. Further, SCT asserts that individuals’ self-efficacy beliefs are influenced by the vicarious experience of socialization agents and that self-efficacy further contributes to an individual’s motivation to take action [15]. Given the key role of social relationships and socialization agents according to SCT, it is not surprising that adolescents with positive social relationships with parents, peers, and teachers benefit from these experiences and, therefore, are more likely to display better social, emotional and behavioral outcomes [17,18,19,20,21]. In the present study, we investigate if and how social relationships with key socialization agents (measured as relationships with parents, bullying victimization via peers, and relationships with teachers) are directly associated with adolescents’ willingness to intervene in bullying, as well as indirectly through self-efficacy in social conflicts. The following research reviews key evidence for the current study.

### 1.1. How Do Social Relationships Influence Adolescents’ Self-Efficacy?

Self-efficacy has been characterized as the “beliefs in one’s capabilities to organize and execute the courses of action required to produce given attainments” by Bandura [15] (p. 3). He explained that self-efficacy stems from four sources: mastery experience (i.e., past accomplishments), vicarious experience (i.e., modeling by others), social and verbal persuasions (i.e., encouragement from parent, peers, teachers) and physiological and emotional states (i.e., stress, anxiety, fatigue, mood). Hence, the development of self-efficacy is influenced by one’s own actions and experiences with parents, peers, and teachers. A high self-efficacy is linked with seeking out challenges and a quick recuperation from failures or defeat; in contrast, low self-efficacy contributes to a tendency to avoid challenges and a higher likelihood of the perception of those as risks as opposed to opportunities. Self-efficacy can be conceptualized broadly in terms of general self-efficacy or more domain-specific, such as academic self-efficacy or self-efficacy in social conflicts [15]. Self-efficacy in social conflicts relates to adolescents’ confidence in their ability to act successfully in social conflict situations and, thus, is an important factor in terms of adolescents’ motivation to intervene in a bullying incident [22].

Because a child’s family is the primary socialization agent, parents, siblings, and other caregivers play a pivotal role in the child’s emotional, social, and cognitive development [15,23,24]. There is some research linking the parent–child relationship to self-efficacy [23,24,25]. Past research has shown that children with good relationships with their parents have positive self-esteem and higher levels of social competence compared to those children who do not [26,27]. Parents that promote effective interactions with the environment also enhance their children’s self-efficacy and competence beliefs [28]. Hence, there is some indication that positive child-parent relationships may be sources for self-efficacy, which is further reflected in positive self-esteem and high levels of social competence [29,30]. However, it is unclear whether a positive parent–child relationship is also linked to children’s self-efficacy in social conflicts.

Relationships with peers are important to adolescents’ development of self-efficacy because peers are a crucial element in adolescents’ socialization and influence their own self-perception [28,31]. The peer environment opens up many possibilities of peer-to-peer learning and peers function as role models [32]. Thus, positive peer relationships characterized by encouragement, sympathy, mutual support, and acceptance might positively influence the development of self-efficacy in social conflicts through providing sources of self-efficacy via vicarious experience and encouragement. Indeed, positive peer relationships have shown to increase adolescents’ self-efficacy in intervening in bullying incidents [33,34]. In contrast, if adolescents experience bullying victimization, this experience may diminish their self-efficacy in social conflicts. They might feel less self-efficacious because they often experience situations where they feel powerless or helpless which negatively impacts their experience of mastery. Past research on victims’ personal characteristics supports the idea that victims lack personal agency by finding that victims are often unassertive, cautious, rarely fight back, and show submissive behavior [35,36,37]. Adolescents who experience social exclusion as one facet of bullying victimization might also miss the opportunity of vicarious experiences to learn from their peers how to deal with social conflicts. Indeed, some research has found that higher levels of bullying victimization predicted lower levels of self-efficacy [38,39].

Teachers often intervene in bullying and are successful in dealing with bullying among students [40,41]. Hence, teachers might be an important source for creating self-efficacy by offering vicarious experiences as social models. A positive teacher–student relationship might also be characterized by an intense teacher–student communication which can influence students’ self-efficacy expectations via encouragement. For example, students who receive positive feedback from their teachers concerning their skills and capabilities to deal with conflictual situation are more likely to persist in the face of setback [28].

### 1.2. How Do Social Relationships Influence Adolescents’ Willingness to Intervene in Bullying?

Although the quality of the parent–child relationship predicts children’s prosocial behavior, sociability levels and prosocial skills in general [42,43,44], the role of the parent–child relationship in adolescents’ behavior as bystander or defender in bullying has been less researched. A possible association between the quality of parent–child relationships and adolescents’ willingness to intervene in bullying can be explained as follows. It is proposed that children who perceive their parents as supportive and their parents additionally serve as role models; thereby increasing the probability that these children also support others in need. Empirical results concerning the association between parent–child relationships and adolescents’ willingness to intervene are ambiguous. In one study, attachment to parents was positively related to the defender status of the child [45]. In another study, parental support did not significantly predict prosocial bystander behavior [46].

Victims of bullying report lower levels of peer support compared with adolescents not involved in bullying [47]. Further, victims of bullying were found to have low social status within the peer group [12,48]. Therefore, victims of bullying might be less likely to intervene in bullying because they fear further victimization without the support of their peers. Some research has demonstrated that peer support is positively linked to prosocial bystander behavior [46]. Other research has found a positive association between being a passive bystander and a victim of bullying [11] as well as a negative association between being an active bystander (defender) and a victim of bullying [49].

Although several studies revealed that classroom or school-wide intervention and prevention programs are needed to deal successfully with bullying (e.g., [41,50,51], only a very few studies investigated how relationships with teachers influence students’ willingness to intervene in bullying. Teachers are important to consider because they are in an powerful position to promote a positive climate within class [51]. In addition, positive teacher–student relationships can increase students’ well-being, psychological health, prosocial behavior, school engagement, academic achievement, and behavioral adjustment [52,53,54,55,56]. It might also be likely that students who experience positive teacher–student relationships are more willing to intervene in bullying because they feel safe at school and believe that their pro-social behavior will be encouraged by their teachers. The important role of teacher–student relationships in bullying is further highlighted by studies showing that positive and supportive teacher–student relationships buffer against the negative consequences of bullying for victims, increases student’s willingness to report bullying and seek help [56,57,58]. Indeed, there is some empirical evidence that teacher support is positively associated with prosocial bystander behavior and positive teacher–student relationships are positively related to students’ willingness to intervene in bullying while conflictual teacher–student relationships were positively linked to being a passive bystander [46,59,60].

### 1.3. How Does Self-Efficacy Influence Adolescents’ Willingness to Intervene in Bullying?

The lack of confidence in one’s capacity to successfully intervene might result in a lower probability of intervention. Thus, it can be assumed that self-efficacy in social conflicts is an important correlate of students’ willingness to intervene in bullying. Indeed, previous research found evidence for a positive association between adolescents’ perceived self-efficacy and their actual or potential intervention in bullying incidents [22,61,62,63,64,65,66]. As stated above, parents, peers, and teachers may provide vicarious experiences as well as social and verbal persuasion in terms of support and encouragement. Social relationships may also enhance adolescents’ experience of mastery by providing support and reduce negative physiological experiences of stress and anxiety. Hence, all three socialization agents can potentially contribute to the sources of self-efficacy postulated by SCT [15]. Thus, it can be assumed that social relationships might not only influence adolescents’ willingness to intervene in bullying directly but also indirectly through self-efficacy in social conflicts.

### 1.4. The Present Study

Although research attention on bystanders of bullying is increasing, the role of interpersonal relationships for both self-efficacy in social conflicts and adolescents’ willingness to intervene in bullying is still not entirely clear. Therefore, the present study had the purpose to investigate the direct and indirect association among relationships with parents, peers, teachers, self-efficacy in social conflicts, and adolescents’ willingness to intervene in bullying. Investigating the links between relationships with parents, peers, and teachers and willingness to intervene in bullying in one study will enable us to compare the magnitude of each of the social relationships on adolescents’ self-efficacy in social conflicts and their willingness to intervene. The findings might contribute to the development of prevention and intervention programs that may at the same time ensure optimal psychological and academic outcomes for bystanders. The following hypotheses were formulated to guide this research:

**Hypothesis 1** **(H1).** Positive relationships with parents and teachers will be directly and positively and bullying victimization via peers will be directly and negatively related to higher levels of self-efficacy in social conflicts.

**Hypothesis 2** **(H2).** Positive relationships with parents and teachers will be directly and positively and bullying victimization via peers will be directly and negatively associated with adolescents’ willingness to intervene in bullying situations.

**Hypothesis 3** **(H3).** Relationships with parents, peers, and teachers will be indirectly related to adolescents’ willingness to intervene in bullying situations via higher levels of self-efficacy in social conflicts.

## 2. Materials and Methods

### 2.1. Participants and Sampling Procedure

A stratified random sample from the Eastern German federal state of Saxony with types of school as strata was used in this study. The probability of a school to be selected was equivalent to the size of students per school (randomized PPS sampling scheme) was applied. Of the 41 schools initially contacted, 24 offered to participate (58.5% school level response rate). No more than three 6th grade classes and three 8th grade classes were recruited for participation, i.e., up to six classes per school. The non-response rate at the student level was 28% (*N* = 581), with 419 not having obtained written parental consent, 109 being absent, 26 were involved in external school projects, eight refused to participate, four attended internships, another four were on vacation, two were unexcused, and another two had not been informed of this study because they had just recently joined the class.

The final sample was composed of 114 school classes from 24 schools, including seven secondary grammar schools, 13 non-academic-track secondary schools, and four schools for children with special needs (e.g., students with learning disabilities, students with emotional and behavioral problems). About equal participation was achieved across 6th grade (*n* = 1080) and 8th grade (*n* = 991) classes, providing a final sample of 2071 participants (48.8% females). Among the sample, the majority (50.4%; *n* = 1044) were in non-academic-track secondary schools, 43.7% (*n* = 904) attended secondary grammar schools, and 5.9% (*n* = 123) attended schools for children with special educational needs. Students were between 12 and 17 years old (*M* = 13.63, *SD* = 1.17), with 20.6% (*n* = 427) 12-year-olds, 27.1% (*n* = 562) 13-year-olds, 23.8% (*n* = 493) 14-year-olds, 24.6% (*n* = 509) 15-year-olds, 3.3% (*n* = 69) 16-year-olds, 0.3% (*n* = 6) 17-year-olds, and 0.2% (*n* = 5) specified no age.

### 2.2. Measures

#### 2.2.1. Dependent Variable

Adolescents’ willingness to intervene in bullying situations. For the purpose of increasing the validity of the measure, a definition of traditional bullying was introduced at the outset of the questionnaire. This definition included the key features of bullying proposed by Olweus [1], i.e., intention to hurt, imbalance of power, and repetition. Adolescents’ willingness to intervene in bullying situations was assessed with two vignettes. These vignettes have been adopted from previous research to assess teachers’ willingness to intervene in bullying [67]. Adolescents’ willingness to intervene in verbal bullying situations was assessed with the initial vignette followed by a vignette assessing willingness to intervene in relational bullying situations (in German language):

Verbal bullying vignette

“You hear a sixth-grade student calling out to another: ‘Nerd, bootlicker, asshole.’ The student tries to ignore the remarks but is obviously sad. You have already observed the same thing a few days ago.”

Relational bullying vignette

“During the break, you hear a sixth-grade student say to another: ‘No, absolutely not. I’ve already told you that you can’t join us.’ The student has no friends in class and is alone throughout the break and looks sad. It’s not the first time that the student was excluded from playing.”

Participants indicated their agreement to statements following each vignette: “When I observe something like that, I interfere and try to stop the behavior.” The participants then rated the items on a five-point ordinal scale: (1) “completely disagree”, (2) “disagree”, (3) “neither agree nor disagree”, (4) “agree”, and (5) “completely agree”. There was a sufficiently high correlation between the response items of the two vignettes—*r* = 0.62, *n* = 2049, and *p* < 0.001.

#### 2.2.2. Independent Variable

Self-efficacy in social conflicts. Three items where combined to a scale measuring self-efficacy in social conflicts (e.g., “I manage to cope well even with difficult classmates” developed by Jerusalem and Klein-Heßling [68]). Response options ranged from (1) “completely disagree” to (4) “completely agree” with higher scores indicating higher self-efficacy in social conflicts (Cronbach’s alpha = 0.68).

Parent–child relationships. Parent–child relationships were measured with four items (Cronbach’s α = 0.89), such as “I have the feeling that I can talk about everything with my father or mother” developed by Fend and Prester [69]. The participants rated on a five-point ordinal scale ranging from 1 (completely disagree) to 5 (completely agree), with higher scores indicating more positive parent–child relationships.

Verbal and relational bullying victimization. Bullying victimization was measured with a scale consisting of three items reflecting verbal and relational bullying victimization, such as “Other students have spread lies and rumors about me and have tried to make me unpopular with others” developed by Olweus [70]. The items were related on a five-point ordinal scale: (1) “never”, (2) “once or twice”, (3) “twice or thrice a month”, (4) “about once a week” or (5) “several times a week”, with higher scores indicating higher bullying victimization (Cronbach’s alpha = 0.72).

Teacher–student relationships. Teacher–student relationships were assessed with four items, such as “Most teachers try to understand the peculiarities and problems of individual students” developed by Fend and Prester [69]. The participants rated on a five-point ordinal scale of 1 (completely disagree) to 5 (completely agree), with higher scores indicating more positive teacher–student relationships (Cronbach’s alpha = 0.77).

#### 2.2.3. Control Variables

Socio-demographic variables. Data were collected on students’ age, sex (1 = male or 2 = female), school type (non-academic-track secondary school [Oberschule], grammar school [Gymnasium]), school for students with special needs [Förderschule] and grade (6th or 8th). Age and sex were entered as control variables as these variables were linked to students’ willingness to intervene in bullying in past research. In particular, younger students and girls were more likely to defend the victim compared to older students and boys [22,34,64,71,72,73].

### 2.3. Procedures

This study was approved by the data protection officer and educational authority of the federal state of Saxony in Germany. Proceeding the approval, 41 schools were invited to this study via email. For those children who were minors, written parental consent was acquired. The duration of this study was from June 2014 to October 2014, and trained research assistants administered the paper-and-pencil questionnaires to students in the classroom during normal school hours. Students were informed that participation was anonymous, voluntary, and that they had the right to withdraw at any time without penalty. Administration time ranged from 30 to 45 min.

### 2.4. Data Analyses

Questionnaire items were averaged across scales to obtain mean scores for each scale. Initial analyses, including descriptive statistics and intercorrelations between variables, were conducted. A structural equation modeling was applied to conduct mediation analysis using as latent variables the parent–child relationship, bullying victimization, the teacher–student relationship, self-efficacy, and adolescents’ willingness to intervene in bullying. The factor loadings of the two observed variables willingness to intervene in a verbal bullying incident and a relational bullying incident were freely estimated to form the latent variable “willingness to intervene in bullying”, with the factor variance fixed to one. Mediation analyses were conducted using *Mplus* 8.3 software [74]. The direct effects of parent–child relationship, bullying victimization, and teacher–student relationships on adolescents’ willingness to intervene in bullying incidents and on self-efficacy in social conflicts were assessed. In addition, we tested whether potential effects of parent–child relationships, bullying victimization, and teacher–student relationships on adolescents’ willingness to intervene in bullying incidents would be mediated via self-efficacy in social conflicts. Maximum likelihood estimation with robust standard errors (MLR) was computed as the distribution of the two dependent variables departed from normality. A bias-corrected bootstrapping procedure with 5000 samples was employed to test for significance of the indirect effects. The issue of missing data was addressed by using full information maximum likelihood (FIML) estimation. The complex sampling option in *Mplus* (complex-option) was used to correct standard errors due to the multi-level structure (i.e., students nested within school classes) of the data. The fit of the model was examined by considering the following indices: The Comparative Fit Index (CFI), the Tucker–Lewis Index (TLI), the Root Mean Square Error of Approximation (RMSEA), and the Standardized Root Mean Square Residual (SRMR). CFI and TLI values of 0.95 or greater and RMSEA and SRMR values of 0.05 or less and are interpreted as evidence of models that fit well [75]. Cohen’s *f*^2^ was used to calculate the effect size of multivariate regression analyses. According to Cohen [76], *f*^2^ ≥ 0.02 represents a small effect size, *f*^2^ ≥ 0.15 a medium effect size, and *f*^2^ ≥ 0.35 a large effect size.

## 3. Results

### 3.1. Descriptive Statistics

Frequency rates of students’ willingness to intervene in verbal and relational bullying are presented in Table 1.

Correlations and descriptive statistics for the study variables were conducted (see Table 2).

### 3.2. Direct and Indirect Associations among Adolescents’ Social Relationships, Self-Efficacy, and Willingness to Intervene in Bullying

We investigated direct effects of parent–child relationships, bullying victimization via peers, and teacher–student relationships on self-efficacy in social conflicts and adolescents’ willingness to intervene in bullying, as well as indirect effects of relationships with parents, peers, and teachers on adolescents’ willingness to intervene in bullying via self-efficacy in social conflicts, while controlling for participants’ age and sex. The model had a good fit: χ^2^ = 236.28, *df* = 94, χ^2^/*df* = 2.51, *p* < 0.001, CFI = 0.98, TLI = 0.98, *RMSEA* = 0.027 [90% CI = 0.023, 0.031], and *SRMR* = 0.02; standardized factor loadings ranged from 0.61 to 0.87. Unstandardized and standardized coefficients are reported in Table 3.

**Figure 1:**
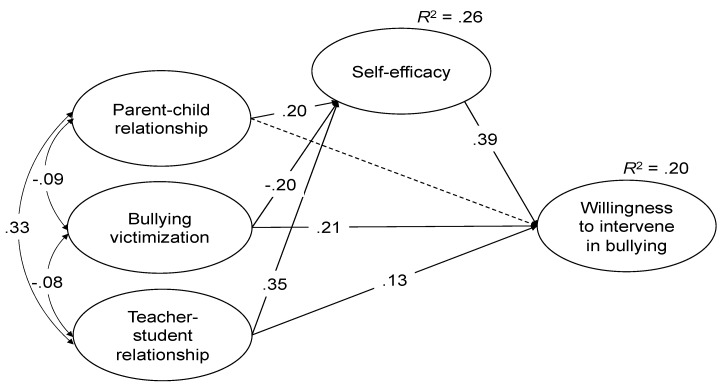
Standardized estimates of relationships between the latent variables, namely parent–child relationship, bullying victimization, teacher–student relationship, self-efficacy, and willingness to intervene in bullying. Notes: Dash arrows: non-significant path coefficient.

Results showed that higher levels of parent–child relationships predicted higher levels of self-efficacy in social conflicts ($\hat{\beta }$ = 0.20, *p* < 0.001), higher levels of bullying victimization predicted lower levels of self-efficacy in social conflicts ($\hat{\beta }$ = −0.20, *p* < 0.001), and higher levels of teacher–student relationships predicted higher levels of self-efficacy in social conflicts ($\hat{\beta }$ = 0.35, *p* < 0.001). In addition, higher levels of self-efficacy in social conflicts predicted higher levels of willingness to intervene in bullying ($\hat{\beta }$ = 0.39, *p* < 0.001). We also found higher levels of bullying victimization predicted higher levels of adolescents’ willingness to intervene in bullying ($\hat{\beta }$ = 0.21, *p* < 0.001). In addition, higher levels of teacher–student relationships predicted higher levels of adolescents’ willingness to intervene in bullying ($\hat{\beta }$ = 0.13, *p* < 0.001). Parent–child relationships were not significantly associated with adolescents’ willingness to intervene in bullying (see Figure 1).

Furthermore, we found three significant indirect effects on adolescents’ willingness to intervene in bullying. Parent–child relationships ($\hat{\beta }$ = 0.08, 95% CI = [0.05, 0.11]), bullying victimization ($\hat{\beta }$ = −0.08, 95% CI = [−0.12, −0.04]), and teacher–student relationships ($\hat{\beta }$ = 0.14, 95% CI = [0.09, 0.18]) predicted higher levels of adolescents’ willingness to intervene in bullying via greater self-efficacy in social conflicts. The SEM explained 26% of variance in self-efficacy in social conflicts (*R*^2^ = 0.26; Cohen’s *f*^2^ = 0.35, indicating a large effect) and 20% of variance in adolescents’ willingness to intervene in bullying (*R*^2^ = 0.20; Cohen’s *f*^2^ = 0.25, indicating a medium effect).

Finally, in order to explore whether beyond the theoretically predicted mediating role self-efficacy may also have acted as a moderator, we conducted a series of supplementary analyses to investigate interactions between parent–child relationships and self-efficacy, bullying victimization and self-efficacy, and teacher–student relationships and self-efficacy when predicting adolescents’ willingness to intervene in bullying. None of the three two-way interactions were significant, hence, potential moderation effects by self-efficacy could be ruled out. More details can be requested from the first author.

## 4. Discussion

The current investigation utilized SCT to understand the direct and indirect associations among relationships with parents, peers, and teachers, self-efficacy in social conflicts, and adolescents’ willingness to intervene in bullying.

We found support for our hypothesis that parent–child relationships, bullying victimization via peers, and teacher–student relationships will be positively directly associated with higher levels of self-efficacy in social conflicts (H1). Our findings are aligned with previous research emphasizing the important role of parents and teachers in the development of adolescents’ academic and general self-efficacy [23,24,25,28]. Furthermore, our results extend previous findings to self-efficacy in social conflicts. Parents and teachers contribute to adolescents’ self-efficacy beliefs by providing sources of self-efficacy in form of social and verbal persuasion via support and encouragement as well as vicarious experiences [15,24,28]. In our analysis, the association with self-efficacy was slightly higher for teacher–student relationships (medium effect) compared to that of parent–child relationships (small to medium effect). It may be that because the items of our measure for self-efficacy in social conflicts was primarily targeted at conflicts in schools, teachers may have been able to offer support and direct vicarious experiences in the school environment more so than parents. The sources of parents’ contribution to adolescents’ self-efficacy may involve emotional support and encouragement but less so in terms of offering vicarious experiences, which may explain the slightly lower effect compared to that of teachers.

Moreover, our findings showed a negative association between bullying victimization via peers and self-efficacy, which is consistent with previous research [38,39,77]. Experiencing bullying victimization may eliminate or negate the experience of mastery in a social conflict and therefore provide a negative source of self-efficacy in social conflicts. If bullying victimization includes social exclusion, it might reverse or inhibit other sources of self-efficacy, such as those of social support and vicarious experiences [15,24]. In sum, our findings suggest that social relationships with key socialization agents provide sources for self-efficacy as proposed by SCT, and that these socialization agents play a crucial role in the development of self-efficacy in adolescents’ social conflicts.

We found partial support for our hypothesis that relationships with parents, peers, and teachers would be directly associated with adolescents’ willingness to intervene in bullying situations (H2). We did not find a direct effect of parent–child relationships on adolescents’ willingness to intervene in bullying which is in accordance with Evans and Smokowski [46] but in contrast to Nickerson et al. [45]. In our analysis, parent–child relationships showed an indirect effect on willingness to intervene via self-efficacy, i.e., self-efficacy fully mediated the effect of parent–child relationships on willingness to intervene. This implies that parent–child relationships affected adolescents’ willingness to intervene by providing sources of self-efficacy via support and encouragement but not by directly influencing the particular behavioral intention itself. Such a finding might be due to parents not being directly involved in the environment where the bullying behavior has taken place (i.e., at school).

We found a positive association between bullying victimization via peers and adolescents’ willingness to intervene in bullying which contrasts to the findings of previous research on bullying victimization [11,49]. Our varying results from past research might be explained by the fact that in the current study we measured behavioral intentions and not actual intervention behaviors. It might be that victims are more likely to be willing to intervene but may be less likely to actually do so if given the chance. Victims can relate to the experience of being bullied and will likely experience an increased sense of empathy with the victim in the hypothetical scenarios included in this study. Indeed, past research has shown that empathy is associated with reported active bystander behavior (defending) [65,78]. However, despite the increased willingness to intervene, past research has also shown that the experience of being victimized may reduce the confidence to carry out the intended intervening behaviors, i.e., victimization has reduced their sense of self-efficacy. Given that self-efficacy is seen as a fundamental factor for an individual’s motivation to take action [15,24], the negative association found between bullying victimization and self-efficacy in social conflicts further supports the suggestion that those who have been victimized may not feel confident to engage in actions to intervene, despite their expressed willingness to do so. The potential tension between an enhanced sense of empathy (increasing the willingness to intervene) and lowered self-efficacy (decreasing the confidence to intervene) may further explain the cognitive dissonance between actual and desired behaviors that bystanders have been shown to experience in previous research [11].

Lastly, we found a positive link between the quality of teacher–student relationships and adolescents’ willingness to intervene which is consistent with previous research [46,59,60]. This finding suggests that teacher–student relationships can enhance adolescents’ initiative for intervening in bullying scenarios beyond the sources of self-efficacy in social conflicts that teachers provide (e.g., vicarious experience, encouragement and support). The impact of positive relationships with teachers to decrease school bullying is consistent with previous findings on the contribution of a general positive school climate to reduce bullying rates [51,79], whereby school climate is typically measured by the quality of teacher–student relationships [80]. Hence, our findings are aligned with propositions of researchers that general positive climate including positive relationships serve as a direct environmental factor to impact positively on reducing school bullying.

We further add to the literature that relationships with parents, peers, and teachers were indirectly related to adolescents’ willingness to intervene in bullying situations via higher levels of self-efficacy (H3). These findings underscore assertions proposed by SCT that relationships with key socialization agents, such as parents, teachers and peers, provide sources of self-efficacy which in turn is crucial for predicting positive adjustment [15,24]. Moreover, our analysis confirms these assertions for self-efficacy in social conflicts and behavior in terms of intervening in bullying scenarios. In sum, our analysis confirms the general assumptions of SCT and the usefulness of applying its approach to social conflicts such as bullying situations.

Our findings confirm suggestions and efforts put forward in terms of systemic intervention approaches that address different social contexts in which the individual is situated [34,41]. Parents, teachers as well as peers have been involved in the successful prevention and intervention of school bullying [81]. Some of these programs have also successfully focused on self-efficacy [82]. Our findings suggest that a specific focus on relationships as well as self-efficacy in social conflicts may add further advantages when implementing those programs. Further, previous evidence and approaches that emphasize the importance of the role of the teacher in this context is confirmed by the current research.

## 5. Limitations and Outlook on Future Research

Some limitations of the current study must be noted. First, the cross-sectional nature of the present investigation makes it impossible to draw causal inferences. To overcome this limitation, longitudinal study designs are required. Second, only self-reports were used to assess the main variables in this study, namely self-efficacy, intervention willingness, and relationships with parents, peers and teachers. Because some students might tend to hide their behavioral intentions about not helping the victim social desirability might be an issue. Future research should combine peer-reported and self-reported bystander behavior, as well as parent, peer, teachers, and self-reported adolescents’ social relationships. Third, in the present study, we measured how adolescents believe they intent to intervene in a given situation, but not necessarily if they have *actually* intervened. Follow-up research should consider actual intervention behavior and compare the findings with the findings of the present study. Fourth, self-efficacy was not measured by a specific self-efficacy scale concerning bullying. Follow-up studies should try to reproduce our findings by using a scale which measures adolescents’ self-efficacy to intervene in social conflicts related to bullying specifically. Fifth, we did not consider potential order effects regarding the presentation of the two bullying vignettes, i.e., there may have been a potential halo effect of willingness to intervene in verbal (1st vignette) to relational (2nd vignette) bullying. Some research has shown that question-order effects influence the measurement of bullying among adolescents [83]. Thus, future research should clarify whether changing the sequence of vignettes might also affect adolescents’ reports on willingness to intervene in bullying. Sixth, we did only include age and sex as control variables and only self-efficacy as an intrapersonal factor. Follow-up research should include more control variables (e.g., socioeconomic status) to increase the generalizability of the findings and more potentially relevant intrapersonal factors (e.g., empathy, moral disengagement) to better understand possible confounders to adolescents’ willingness to intervene in bullying. Further, whilst the sample selection method ensured proportionate representations of different types, sizes and locations of schools, rendering the findings as generalizable for the sampling frame (the state of Saxony in Germany), the sample is limited in terms of its cultural and ethnic diversity; hence, replications in other contexts outside of Germany may contribute to the validity of the findings. Finally, we did not measure adolescents’ willingness to intervene in physical or cyber bullying but only adolescents’ willingness to intervene in verbal and relational bullying scenarios. Therefore, future follow-up studies should also consider physical and cyber bullying scenarios to understand adolescents’ intervention willingness in a broader range of bullying incidents. In addition, adolescents’ willingness to intervene may take many forms beyond trying to stop the behavior directly (i.e., console the victim after the bullying episode is over, asking a teacher for help). Future research should include these alternative forms of intervention behaviors.

In terms of linking our findings to SCT, even though our study’s findings show results expected by the sources for self-efficacy explained by Bandura [15], we did not assess those sources directly. Future research is needed to assess whether relationships with parents, teachers, and peers affect self-efficacy in social conflicts via the postulated vicarious experiences as well as social and verbal persuasion. It would also be of interest to ascertain which relationship works primarily via the postulated sources as a mechanism in affecting self-efficacy in social conflicts. Further, we proposed that bullying victimization may result in cognitive dissonance via the conflicting outcomes of increased empathy, increasing a bystander’s willingness to intervene, and reduced self-efficacy, reducing bystander intervention. Empathy was not assessed in the current study; hence, more research is needed to directly assess the interplay of empathy and self-efficacy in behavioral intentions to defend a victim. Lastly, bullying victimization was an inverse of the actual quality of peer relationships. It should be considered whether a positive measure of peer relationships (e.g., number and quality of friendships) would yield comparable results.

## 6. Conclusions

The present study applied SCT to investigate how the perceived quality of relationships with parents, peers, and teachers were directly and indirectly associated with adolescents’ self-efficacy in social conflicts and willingness to intervene in bullying. In accordance with our hypotheses, quality of relationships was significantly associated with higher levels of self-efficacy, and an increased willingness to intervene in bullying. Further, quality of relationships was indirectly associated with adolescents’ willingness to intervene through self-efficacy. Given that the active involvement of students in support of the victim is linked with the termination of bullying events, and the negative outcomes of witnessing bullying for the bystander, it seems to be vital to find ways to increase adolescents’ quality of relationships and self-efficacy in social conflicts to handle bullying successfully. We thus conclude that: (1) efforts to increase students’ willingness to intervene in bullying should promote students’ confidence in dealing with social conflicts and interpersonal relationships with parents, peers, and teachers.; (2) adolescents who experience bullying victimization should be supported in developing self-efficacy because they do not feel confident to engage in actions to intervene, despite their expressed willingness to do so, and (3) self-efficacy plays an important role in understanding the associations between adolescents’ social relationships and willingness to intervene in bullying. Future studies should focus on developing a better understanding of how adolescents can be supported to intervene in bullying.

## Figures and Tables

**Table 1:** Frequency rates of students’ willingness to intervene in verbal and relational bullying.

Variables	Completely Disagree		Disagree		Neither Agree nor Disagree		Agree		Completely Agree	
	%	*n*	%	*n*	%	*n*	%	*n*	%	*n*
Intervention willingness in verbal bullying	20.9	428	22.4	458	34.9	717	16.4	337	5.4	110
Intervention willingness in relational bullying	22.6	463	25.3	518	33.4	684	13.4	274	5.2	107

**Table 2:** Correlations and descriptive statistics among main study variables.

Variables	1	2	3	4	5
Intervention willingness in verbal and relational bullying	—				
Self-efficacy in social conflicts	0.26 **	—			
Parent–child relationships	0.12 **	0.25 **	—		
Bullying victimization	0.11 *	−0.17 *	−0.08 **	—	
Teacher–student relationships	0.21 **	0.30 **	0.27 **	−0.05 **	—
Mean	2.58	2.81	3.94	1.40	3.50
SD	1.02	0.57	0.99	0.71	0.78

Note. * *p* < 0.1; ** *p* < 0.01.

**Table 3:** Unstandardized coefficients (*B*), standardized coefficients ($\left(\hat{\beta }\right)$, and standard errors (in parentheses) for the mediation model.

Parameter Estimates	B (SD)	$\hat{\beta }$ (SD)
Direct Effects		
Parent–child relationship -> Self-efficacy	0.12 (0.02) ***	0.20 (0.04) ***
Bullying victimization -> Self-efficacy	−0.13 (0.02) ***	−0.20 (0.04) ***
Teacher–student relationship -> Self-efficacy	0.29 (0.04) ***	0.35 (0.04) ***
Parent–child relationship -> Willingness to intervene	−0.01 (0.04)	0.01 (0.03)
Bullying victimization -> Willingness to intervene	0.27 (0.06) ***	0.21 (0.03) ***
Teacher–student relationship -> Willingness to intervene	0.24 (0.06) ***	0.13 (0.04) ***
Self-efficacy -> Willingness to intervene	0.84 (0.12) ***	0.39 (0.04) ***
Indirect Effects		
Parent–child relationship -> Self-efficacy -> Willingness to intervene	0.10 (0.02) ***	0.08 (0.02) ***
Bullying victimization -> Self-efficacy -> Willingness to intervene	−0.11 (0.03) ***	−0.08 (0.02) ***
Teacher–student relationship -> Self-efficacy -> Willingness to intervene	0.25 (0.05) ***	0.14 (0.02) ***

Note. *** *p* < 0.001.
